# Volumetric parameters derived from FLT-PET performed at completion of treatment predict efficacy of Carbon-ion Radiotherapy in patients with locally recurrent Nasopharyngeal Carcinoma

**DOI:** 10.7150/jca.46490

**Published:** 2020-10-17

**Authors:** Jiyi Hu, Zhongyi Yang, Jing Gao, Weixu Hu, Jing Yang, Xianxin Qiu, Yingjian Zhang, Guang Ma, Lin Kong, Jiade J. Lu

**Affiliations:** 1 Department of Radiation Oncology, Shanghai Proton and Heavy Ion Center, Shanghai 201321, China.; 2 Department of Radiation Oncology, Shanghai Proton and Heavy Ion Center, Fudan University Cancer Hospital, Shanghai 201321, China.; 3 Shanghai Engineering Research Center of Proton and Heavy Ion Radiation Therapy.; 4 Department of Nuclear Medicine, Fudan University Shanghai Cancer Center, China.; 5 Department of Oncology, Shanghai Medical College, Fudan University, China.; 6 Center for Biomedical Imaging, Fudan University, China.; 7 Shanghai Engineering Research Center of Molecular Imaging Probes, China.

**Keywords:** locally recurrent nasopharyngeal carcinoma, 3'-deoxy-3'-[18F]fluorothymidine, outcome prediction

## Abstract

The purpose of this study was to investigate the role of 3'-deoxy-3'-[^18^F]fluorothymidine (FLT)-PET for predicting the outcome of patients with locally recurrent nasopharyngeal carcinoma (LR-NPC) treated by carbon-ion radiotherapy (CIRT). Patients received FLT-PET/CT scan one-week prior to or after completion of CIRT were enrolled in the study. All patients were from prospective trials or treated using a standardized protocol. Time-dependent receiver operator characteristics (ROC) were used to determine the optimal cutoff values for FLT-PET parameters. Univariable and multivariable analyses of local progression-free survival (LPFS) were performed using Cox regression, to examine the prognostic value of FLT-PET parameters, including SUV_max_, metabolic tumor volume (MTV) and total lesion thymidine (TLT). A total of 41 patients were enrolled. Elevated MTV and TLT were significantly associated with worse LPFS, in both univariable and multivariable analyses. ROC analysis revealed that both an MTV value higher than 8.6 and a TLT value higher than 14.9 were predictive of increased risk of developing local recurrence, the adjusted HRs were 5.59 (*p*=0.009) and 7.76 (*p*=0.002), respectively. In conclusion, FLT-PET was found to be a promising prognostic tool for LR-NPC patients and might play a role in the treatment guidance.

## Introduction

Nasopharyngeal carcinoma (NPC) is endemic in Southeast Asia and approximately 10-15% of the patients may develop locoregional recurrence after definitive intensity modulated radiation therapy (IMRT). Re-irradiation is currently the mainstay of treatment for patients with locoregionally recurrent NPC (LR-NPC); however, re-irradiation for LR-NPC is clinically challenging. LR-NPC is a group of highly heterogeneous malignancies 1-3, with locoregional control ranging from several months to years. A subgroup of the patients may have significantly worse outcome because of the resistance of the recurrent tumor cells 4. Meanwhile, re-irradiation with a combined dose above 100 Gy from two courses of radiotherapy was usually associated with severe adverse effects (SAE) 5. Because of its biological and physical advantages 6, re-irradiation by carbon-ion radiotherapy (CIRT) is becoming a promising treatment option for LR-NPC. Our previous study has demonstrated that CIRT could provide satisfactory survival rates with infrequent toxicities for LR-NPC patients 7. However, the optimal dose of CIRT may vary from patient to patient. A tailored dose could provide potentially higher local control for patients with high-risk of treatment failure, while maintain the incidence of SAE at an acceptable level for the rest of the patients. Therefore, a reliable marker is in need to identify patients with worse outcomes.

[^18^F]fluorodeoxyglucose (FDG)-positron emission tomography/computer tomography (PET/CT), as a technique of metabolic imaging, was widely used to predict the treatment response for various malignancies 8-11. However, interpretation of FDG-PET/CT could be confounded by false positive readings caused by peritumor inflammation and physiological changes 12, 13.

Recently, 3'-deoxy-3'-[^18^F]fluorothymidine (FLT), a thymidine analog, was increasingly used as a new tracer to image the proliferation of cancers 14. FLT is trapped within cells, after it's taken up and phosphorylated by thymidine kinase 1 (TK1) 15, 16. Tk1 is a salvage enzyme that has an increased activity during the S phase of the cell cycle 17. Therefore, FLT uptake is closely related to cellular proliferation. In comparison to FDG, FLT may distinguish tumor proliferation from radiotherapy-induced inflammation and physiologic changes, thus more effectively predict the outcome of patients with head and neck cancer after radiotherapy 18. Clinically, FLT-PET was shown to be associated with survivals of various malignancies 19-23. However, the prognostic role of FLT-PET was not previously examined in patients with LR-NPC.

The objective of this study was to examine the role of parameters derived from FLT-PET scan performed at the completion of treatment in predicting disease recurrence in patients with LR-NPC.

## Materials and Methods

### Patients and treatment

Between June 2015 to January 2018, LR-NPC patients who received FLT-PET/CT scan one week prior to or after completion of CIRT were enrolled in this study. And a patient would be deemed eligible if he or she 1) had pathologically confirmed WHO type I/II/III primary nasopharyngeal carcinoma; 2) completed a definitive course of radiotherapy to a total dose of ≥ 66Gy at least 6 months ago; 3) was diagnosed of LR-NPC by pathology or imaging study; 4) aged 18 to 80 years; 5) had no distant metastasis; 6) had not received any other types of local treatment after recurrence; 7) had a Karnofsky performance score ≥ 70; 8) was willing to accept adequate contraception for women with childbearing potential; 9) was willing to sign the written informed consent. Patients that received concurrent chemotherapy were excluded from this study.

All patients enrolled were treated prospectively according to either one of the 2 ongoing phase I/II clinical trials (NCT02569788, NCT02795195) or the standardized treatment protocol used at Shanghai Proton and Heavy Ion Center. Patients would receive 50 to 65GyE (at 2GyE/daily fraction to 3GyE/daily fraction) if they were treated using the standardized protocol. And patients would receive 57.5-65GyE at 2.5GyE/daily fraction, or 54-63GyE at 3GyE/daily fraction respectively, if they were enrolled into one of the trials. Patients with locally advanced disease were recommended to receive induction chemotherapy prior to CIRT with cisplatin-containing regimen.

This study was approved by the institutional review board at Shanghai Proton and Heavy Ion Center (SPHIC). Informed consent was obtained from all patients. All methods were performed in accordance with the Declaration of Helsinki.

### FLT-PET/CT imaging procedures

**Figure 1:**
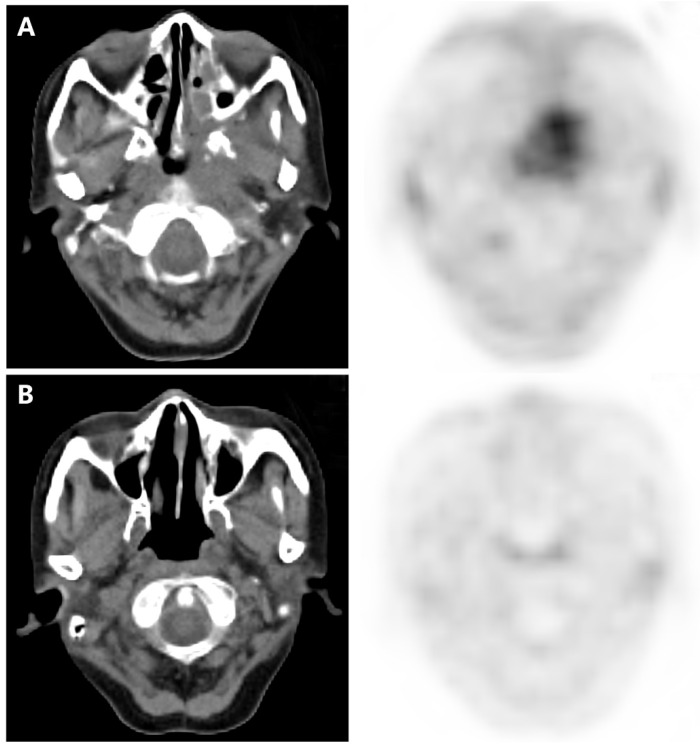
Examples of FLT-PET/CT scans performed at completion of treatment. (**A**) A rT4N0M0 patient who received FLT-PET/CT scan 4 days after completion of CIRT. Significant FLT uptake was observed, and the SUVmax, MTV and TLT were 2.8, 27.7 and 46.2, respectively. This patient developed local recurrence 6 months after completion of CIRT. (**B**) A rT3N0M0 patient who received FLT-PET/CT scan 1 day before completion of CIRT. This patient had no residual FLT uptake and remained complete remission till the last follow-up (23 months after completion of CIRT).

According to the inclusion criteria, all enrolled patients received FLT-PET/CT scan at the completion of CIRT, which was defined to be performed one week prior to or after completion of CIRT. Images of FLT-PET/CT for patients with LR-NPC are illustrated in **Figure 1.**

FLT-PET/CT was performed using a Siemens biograph 16HR PET/CT scanner (Knoxville, Tennessee, USA). Scanning was initiated 1h after administration of the trace (dosage: 7.4 MBq/kg). The trans-axial intrinsic spatial resolution was 4.1 mm (full-width at half-maximum) in the center of the field of view. The data acquisition procedure was as follows: emission images of 3-4 bed positions from head to base of lung were recorded, with 2-3 min per bed position in 3-dimensional mode. CT images were acquired for anatomic correlation and attenuation correction using 120kV, 80~250mA, pitch 3.6, rotation time 0.5 and no contrast agent was allowed.

### FLT-PET imaging interpretation

After reconstruction, quantification of proliferative activity was obtained using the standardized uptake value (SUV) corrected for injected dose, tracer decay, and patient body weight. The maximum SUV (SUV_max_) was recorded. The metabolic tumor volume (MTV) was calculated using a semiautomatic delineation based on 40% of the SUV_max_, and further assessed by two independent nuclear medicine physicians on visual comparison. The total lesion thymidine (TLT) was calculated as the product of MTV and mean SUV of the volume.

### Statistical analysis

The treatment efficacy was assessed using local progression-free survival (LPFS), defined as the time frame from the date of diagnosis of LR-NPC to the date of documented local progression or the last follow-up, whichever came first. Local progression was determined by MRI using the Response Evaluation Criteria in Solid Tumors (RECIST) (version 1.1).

The optimal cutoff values of FLT-PET parameters were obtained per time-dependent receiver operating characteristic (ROC) analysis by maximizing the Youden's index. FLT-PET parameters were categorized by the optimal cutoff values. And LPFS and overall survival were then estimated using Kaplan-Meier method. Univariable and multivariable analyses by Cox proportional hazards model were used to further evaluate the prognostic values of FLT-PET parameters both as continuous variables and categorical variables. Because only 1 patient died during the study period, univariable and multivariable analyses were performed only on LPFS, but not OS.* P* values <.05 (2-sided tests) were considered to be statistically significant. All statistical analyses were conducted using the R statistical software (version 3.4.3).

## Results

### Study population and baseline characteristics

Between June 2015 and January 2018, 41 patients met the inclusion criteria were included in the study. All patients were treated prospectively by CIRT, and 23 patients received cisplatin-containing induction chemotherapy. The baseline characteristics and treatment modalities are detailed in **Table 1.** Among the patients, about 70% had locally advanced disease; all patients except for 1 received IMRT as the first course of radiotherapy for primary disease. Eighteen patients were treated according to the standardized protocol with a median dose of 57.5GyE (range, 50-65GyE) at either 2.5GyE (10 patients) or 3GyE (8 patients) per fraction; 7 and 16 patients were enrolled into one of our phase I/II clinical trials and treated at 2.5GyE per fraction (median dose, 57.5GyE; range, 57.5-60GyE) or at 3GyE per fraction (median dose, 63GyE; range, 54-63GyE).

### The predictive value of FLT-PET volumetric parameters

The median values of SUV_max_, metabolic tumor volume (MTV) and total lesion thymidine (TLT) were 2.2 (range: 0-5.9), 6.2 (range: 0-36.5) and 8.9 (range: 0-55.1), respectively (**Table 2**).

With a median follow-up time of 15.2 months (range: 2.8 to 43.1 months), 16 patients developed local recurrence after CIRT, and only 1 patient died during the study period. The 12-month and 18-month LFPS were 86.0% (95% CI: 75.3%-98.2%) and 71.6% (95% CI: 57.0%-90.1%).

When analyzed as continuous variables, higher MTV and higher TLT were both significantly associated with increased risk of local recurrence, the corresponding HR were 1.10 (95% CI: 1.03-1.18; *p*=0.006) and 1.08 (95% CI: 1.02-1.13; *p*=0.005), respectively (**Table 3**). The prognostic values of those two volumetric parameters were confirmed in multivariable analysis, and the HR were 1.14 (95% CI: 1.05-1.24; *p*=0.002) for MTV and 1.08 (95% CI: 1.02-1.15; *p*=0.006) (**Table 4**).

**Figure 2:**
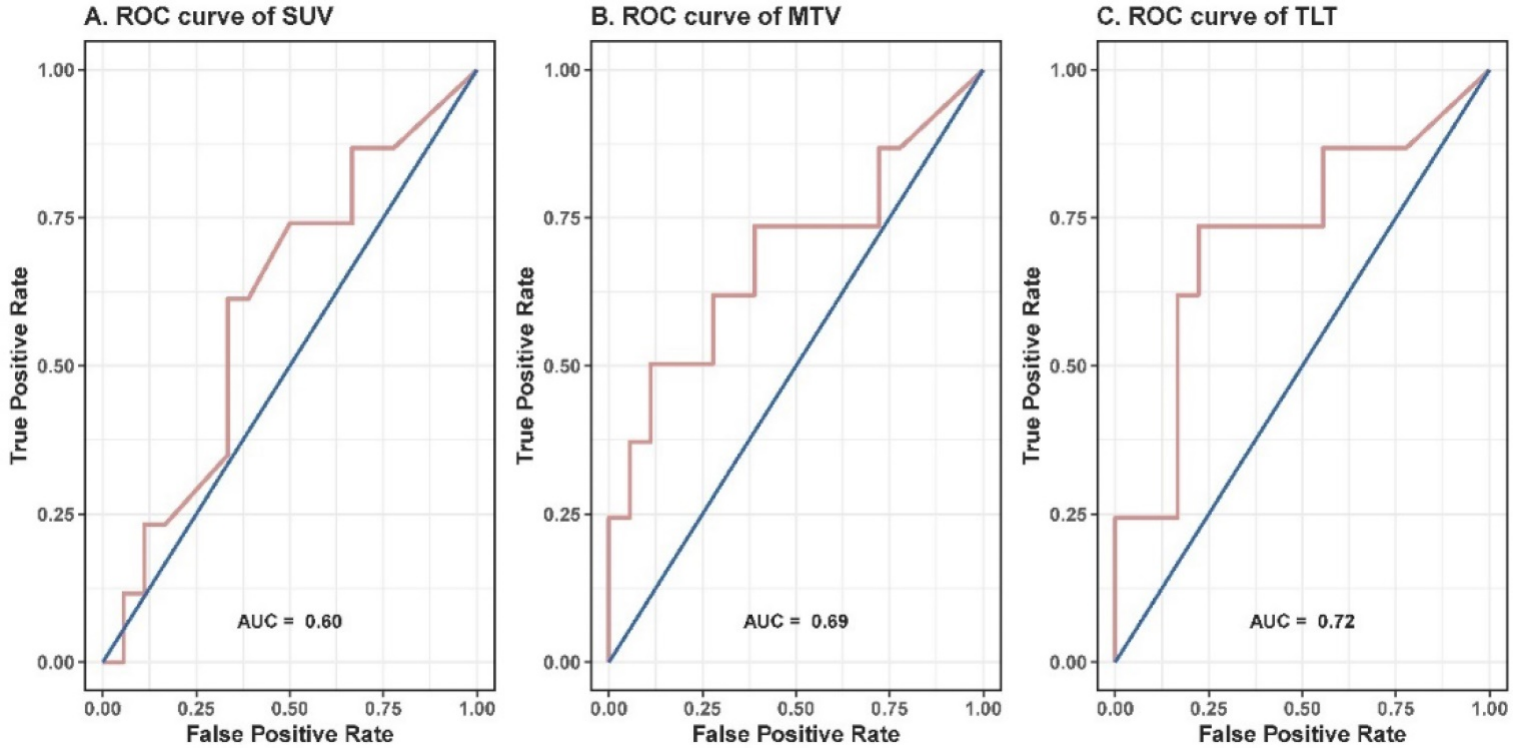
ROC curves of SUV_max_ (**A**), MTV (**B**) and TLT (**C**). The corresponding cutoff values for SUV_max_, MTV and TLT were 2.5, 8.6 and 14.9, and the areas under curve were 0.60, 0.69 and 0.72, respectively.

**Figure 3:**
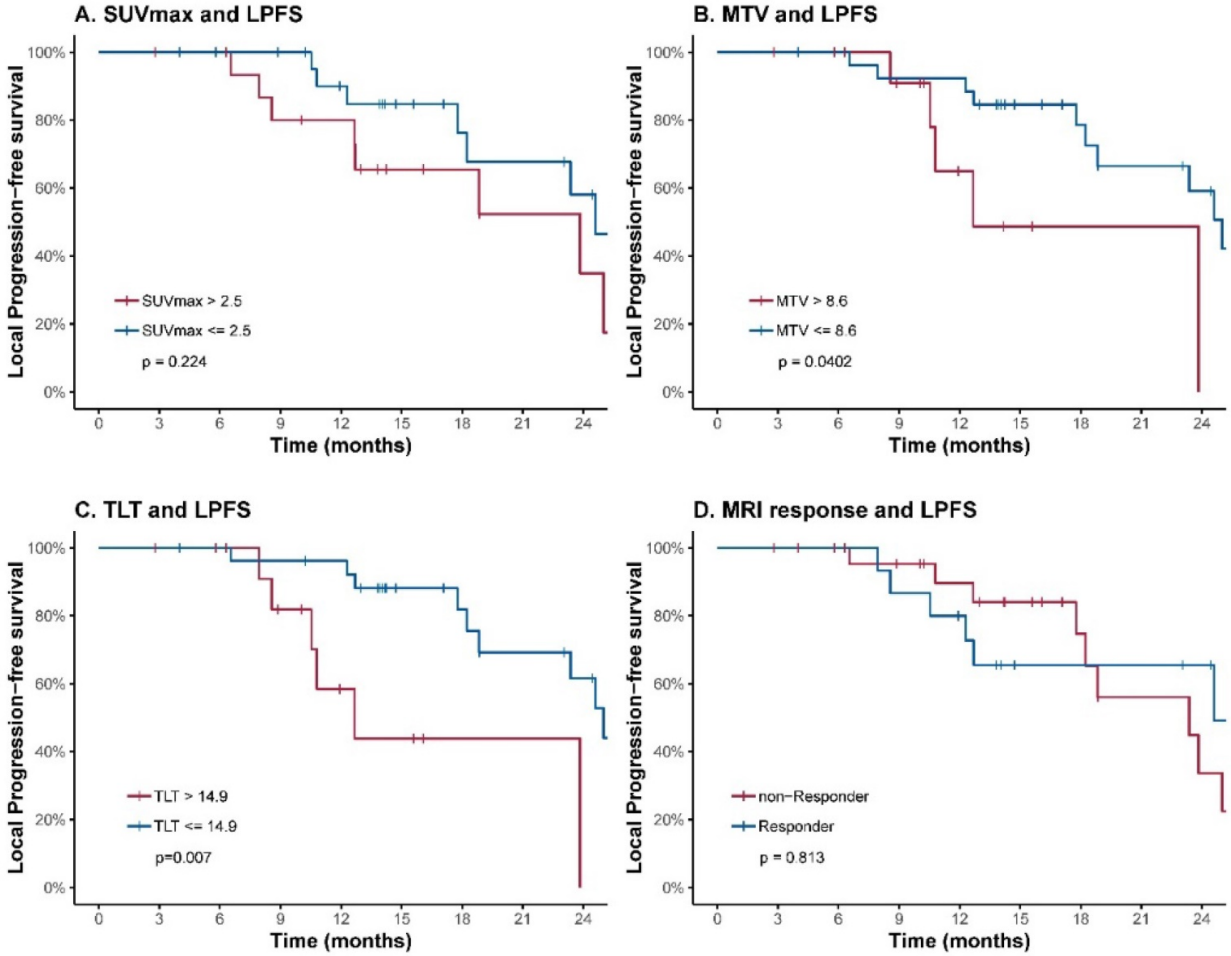
LPFS curves of LR-NPC patients. Patients are stratified according to cutoff values of SUV_max_ (**A**), MTV (**B**), TLT (**C**) and response evaluated by MRI at completion of treatment (**D**). The corresponding cutoff values of FLT-PET parameters were determined by ROC analysis.

To further evaluate the prognostic value of the volumetric parameters, time-dependent ROC analysis was used to determine the optimal cutoff values. The corresponding cutoff values and areas under curve were 8.6 and 0.69 for MTV, 14.9 and 0.72 for TLT (**Table 2, Figure 2**). The univariable analysis showed that patients with an MTV>8.6 (HR: 3.41; 95% CI: 1.06-11.00; *p*=0.040) or a TLT>14.9 (HR: 4.88; 95% CI: 1.56-15.29; *p*=0.007) had significantly worse local control (**Table 3, Figure 3**). The 1-year LPFS was 64.9% (95% CI: 39.2%-100%) vs. 92.3% (95% CI: 82.6%-100%) for patients with MTV>8.6 or not, and 58.4% (95% CI: 33.9%-100%) vs. 96.2% (95% CI: 89.0%-100%) for patients with TLT>14.9 or not. Similarly, in multivariable analysis, the risk of developing local recurrence was significantly higher in patients with MTV>8.6 (HR: 5.59; 95% CI: 1.55-20.15; *p*=0.009) and patients with TLT>14.9 (HR: 7.76; 95% CI: 2.12-28.49; *p*=0.002) (**Table 4**).

In contrast, SUV_max_ and response evaluated by MRI at completion of treatment were not predictive of LPFS.

## Discussion

Salvaging re-irradiation for LR-NPC patients who failed first course of definitive RT is challenging. Recurrence might be caused by radioresistant tumor cells that survived the first course of definitive RT with a dose around 70 Gy. Thus, the disease control could be suboptimal after re-irradiation with a dose similar to or less than 70 Gy. Although higher dose may better control the tumor growth, it is associated with severer radiation-induced toxicities. Patients who receive a combined dose above 100 Gy from initial RT and re-irradiation may have a higher chance of developing SAEs including cranial neuropathy, brain injury, mucosal necrosis and massive hemorrhage, which could result in significantly reduced quality of life, or even death. Because most of its energy is deposited in the region called “Bragg peak”, CIRT is able to accurately deliver high-dose radiation to the tumors while sparing surrounding organs 6, 24. Meanwhile, the higher relative biological effectiveness of accelerated carbon-ion beams enable more effective elimination of resistant tumors cells frequently appeared in LR-NPC 25, 26. Recently, we summarized the initial experience of CIRT for patients with LR-NPC 7. Our results showed an encouraging outcome with a 1-year OS of 98.1% and a 1-year LPFS of 86.6%, whereas severe toxicities were infrequent. Although CIRT could be a promising treatment option for LR-NPC, some of the patients developed local failure shortly after treatment. For those patients, intensified treatment is indicated. Therefore, a robust and clinically feasible marker is in need to identify those high-risk patients and implement individualized treatment.

In the current study, we examined the prognostic roles of parameters derived from FLT-PET performed at completion of treatment for LR-NPC patients. All parameters were evaluated as continuous variables and dichotomized variables (by cutoff values determined by ROC analysis) in both univariate and multivariate analyses. The results showed volumetric parameters including MTV and TLT were predictive of LPFS. Based on ROC analysis, an MTV value higher than 8.6 and a TLT value higher than 14.9 were significantly associated with increased probability of local failure in both univariate and multivariate analyses.

FLT-PET has been used to predict the outcomes of various malignancies, including head and neck cancers treated by radiotherapy 23, 27-34. The prognostic value of FLT-PET in head and neck squamous cell carcinoma (HNSCC) was demonstrated in previous studies. In a study of 28 HNSCC patients, Kishino et al. showed that patients with residual accumulation on post-treatment FLT-PET had significantly worse 3-year local control, though the role of SUV_max_ and volumetric parameters were not examined 32. Hoeben et al. showed in a prospective study that decrease of SUV_max_ and gross tumor volumes segmented by visual delineation on FLT-PET were predictive of 3-year disease-free survival and 3-year locoregional control, respectively 23. The association between FLT-derived parameters and disease control in HNSCC patients was further examined in another prospective study of 53 patients 30. The results showed that pre-treatment total lesion proliferation was significantly correlated to locoregional control, as were SUV_max_ and MTV to OS. Among those studies on HNSCC, only 3 NPC patients were included. Recently, a study of 22 patients with primary NPC was conducted to assess the role of FLT-PET in monitoring treatment response 34. The authors suggested that FLT-PET/CT performed before and after induction chemotherapy prior to chemoradiotherapy might play a role in monitoring the tumor regression; however, its prognostic value was not examined because of the limited sample size and absence of recurrence or death due to the well prognosis of primary NPC. Compared to primary NPC, patients with LR-NPC have significantly worse outcomes and should be regarded as a distinct group of patients. CIRT, as a recently emerging radiation technology, may cause different appearance on PET images. Inubushi and colleagues showed that FLT-PET could be used to predict the outcome of patients with head and neck melanoma treated by CIRT 29; however, the power of the study was limited by its small sample size of 13 patients. To the best of our knowledge, the current study is the first one to examine the association between FLT-PET derived parameters and local control of LR-NPC patients treated by CIRT.

In our study, volumetric parameters (MTV and TLT) showed superiority in predicting LPFS, compared to SUV_max_. It could be partially because volumetric parameters have incorporated more information including tumor volume and total metabolic activity. Similar findings were reported by some of the previous studies. In a study of 32 patients with HNSCC, Hoshikawa et al. showed that metabolic tumor volume and total lesion proliferation based on pretreatment FLT-PET were significantly associated with locoregional control and overall survival, while SUV_max_ played no prognostic role 28. In another study of 26 high-grade glioma patients, Idema et al. showed that both FLT-derived proliferative volumes adapted by the signal-to-background ratio (PV_SBR_) and SUV_max_ were predictive of OS in univariate analysis, but only PV_SBR_ remained statistically significant in multivariate analysis 35.

The optimal timing of FLT-PET scan in terms of predicting survivals and locoregional control of radiotherapy is not clear. Previous studies have examined the role of FLT-PET performed before, during or after treatment. In the study of 28 HNSCC patients conducted by Kishino et al., the results showed the performance of post-treatment parameters tended to improve, compared to those during RT, though not significantly^32^. Similar findings were also found in some studies examining the role of FDG-PET 9, 36, 37. While Inubushi et al. showed that pre-CIRT but not post-CIRT SUV_max_ was associated with significantly improved OS and local control, though this study was limited by its small sample size 29. Theoretically, FLT-derived parameters at completion of treatment may contain valuable information on tumor sensitivity to radiotherapy and provide potentially more accurate prediction of the outcome, compared to baseline parameters.

One strength of this study was that a relatively large number of patients were included. Second, among those, 24 patients were from phase I/II clinical trials, and the rest were treated by prospectively designed protocols. The strict inclusion criteria and prospective treatment design lend power to carefully examine the predictive value of FLT-derived parameters.

A few limitations should be addressed. First, standardized calculation method across centers is required, before volumetric parameters could be widely used to guide daily clinical practice. Second, cutoff values obtained in the current study were not validated. Imaging biomarkers will be more reliable and can potentially be used in clinical practice after being validated with an independent cohort. To find an external cohort for validation, we have queried several major imaging databases. Unfortunately, no patients with recurrent nasopharyngeal carcinoma were identified. Validation of our results by an independent cohort would be ideal; however, although validation was not performed, our study demonstrated the potential predictive value of FLT-PET for patients with rNPC that warrants further investigation. With more rNPC patients receiving FLT-PET scan at our center, we may be able to validate its predictive value in a larger cohort in the future.

## Conclusions

The current study demonstrated that volumetric parameters derived from FLT-PET at completion of treatment could predict the LPFS of LR-NPC patients treated by salvage CIRT. Further validation is needed before those parameters can be used to guide clinical treatment.

## Figures and Tables

**Table 1:** Baseline characteristics and treatment modalities

Characteristics	No. (%)
**Gender**	
Male	27 (65.85%)
Female	14 (34.15%)
**Age at local recurrence**	
Median (range), years	46 (29-70)
<60 years	34 (82.93%)
≥60 years	7 (17.07%)
**Initial radiation technique (IMRT)**	
Non-IMRT	1 (2.44%)
IMRT	40 (97.56%)
**Stage at recurrence**	
I and II	13 (31.71%)
III and IVa/b	28 (68.29%)
**Total dose of re-CIRT**	
Median (range), GyE	60 (50-65)
< 60 GyE	20 (48.78%)
≥ 60 GyE	21 (51.22%)
**Fractionation of re-CIRT**	
Median (range), GyE	3.0 (2.5-3.0)
<3 GyE	17 (41.46%)
3 GyE	24 (58.54%)
**Induction chemotherapy**	
No induction chemotherapy	18 (43.9%)
Induction chemotherapy	23 (56.1%)
**Treatment protocols**	
Standard treatment protocol	18 (43.90%)
Phase I/II trial of CIRT (2.5GyE per fraction)	7 (17.07%)
Phase I/II trial of CIRT (3GyE per fraction)	16 (39.02%)

**Table 2:** Parameters derived from FLT-PET

Parameter	Median	Range	ROC cutoff value	AUC
SUV_max_	2.2	0-5.9	2.5	0.60
MTV	6.2	0-36.5	8.6	0.69
TLT	8.9	0-55.1	14.9	0.72

**Table 3:** Univariable analysis of LPFS

Parameter	HR (95% CI)	*p*
SUV_max_ (as continuous variable)	1.18 (0.81-1.72)	0.387
MTV (as continuous variable)	1.10 (1.03-1.18)	0.006
TLT (as continuous variable)	1.08 (1.02-1.13)	0.005
**SUV_max_ (≤2.5 as ref.)**		
>2.5	1.84 (0.69-4.93)	0.224
**MTV (≤8.6 as ref.)**		
>8.6	3.41 (1.06-11.00)	0.040
**TLT (≤14.9 as ref.)**		
>14.9	4.88 (1.56-15.29)	0.007
**MRI, responder as ref.**		
Non-responder	1.13 (0.41-3.08)	0.813
**Gender, female as ref.**		
Male	0.62 (0.22-1.80)	0.384
**Age, <60 year-old as ref.**		
≥60 year-old	0.36 (0.05-2.71)	0.318
**Stage, stage I/II as ref.**		
Stage III/IV	1.81 (0.51-6.44)	0.359
**Induction Chemotherapy, without chemotherapy as ref.**		
With chemotherapy	1.97 (0.68-5.72)	0.213
**Dose to GTV, <60 GyE as ref.**		
≥60GyE	0.65 (0.20-2.05)	0.460
**Fractionation, <3 GyE per fraction as ref.**		
3GyE per fraction	0.48 (0.15-1.52)	0.209

**Table 4:** Multivariable analysis of LPFS

Parameter	HR (95% CI)	*p*
SUV_max_ (as continuous)	1.27 (0.79-2.05)	0.327
MTV (as continuous)	1.14 (1.05-1.25)	0.002
TLT (as continuous)	1.08 (1.02-1.15)	0.006
**SUV_max_ (≤2.5 as ref.)**		
>2.5	2.27 (0.73-7.09)	0.157
**MTV (≤8.6 as ref.)**		
>8.6	5.59 (1.55-20.15)	0.009
**TLT (≤14.9 as ref.)**		
>14.9	7.76 (2.12-28.49)	0.002
